# Explaining the gender gap in COVID-19 vaccination attitudes

**DOI:** 10.1093/eurpub/ckad052

**Published:** 2023-05-13

**Authors:** Dimiter Toshkov

**Affiliations:** Institute of Public Administration, Leiden University, The Hague, The Netherlands

## Abstract

**Background:**

Women have been significantly more likely than men to express hesitancy toward COVID-19 vaccination and, to a lesser extent, to refuse vaccination altogether. This gender gap is puzzling because women have been more likely to perceive higher risks from COVID-19, to approve more restrictive measures to fight the pandemic and to be more compliant with such measures.

**Methods:**

This article studies the gender gap in COVID-19 vaccination attitudes using two nationally representative surveys of public opinion fielded in February 2021 and May 2021 in 27 European countries. The data are analyzed using generalized additive models and multivariate logistic regression.

**Results:**

The data analyses show that hypotheses about (i) pregnancy, fertility and breastfeeding concerns, (ii) higher trust in Internet and social networks as sources of medical information, (iii) lower trust in health authorities and (iv) lower perceived risks of getting infected with COVID-19 cannot account for the gender gap in vaccine hesitancy. One explanation that receives support from the data is that women are more likely to believe that COVID-19 vaccines are unsafe and ineffective and this leads them to perceive the net benefits of vaccination as lower than the associated risks.

**Conclusions:**

The gender gap in COVID-19 vaccine hesitancy results to a large extent from women perceiving higher risks than benefits of the vaccines. While accounting for this and other factors decreases the gap in vaccine hesitancy, it does not eliminate it completely, which suggests further research is needed.

## Introduction

With the spread of the COVID-19 pandemic, significant differences between men and women emerged in relevant attitudes and behaviors. In particular, women were found to be less likely to declare intentions to get vaccinated against COVID-19, across very different national contexts,^1–9^ as confirmed by a recent systematic review and meta-analysis as well.^10^ This is puzzling because it contrasts with the more cautious approach toward the pandemic exhibited by women, who were more likely to consider the pandemic a very serious health problem, to approve and to comply with restrictive measures imposed by governments to fight the pandemic.^11^ Moreover, women are supposed to be more risk averse than men in general,^12^ which would lead us to think that they would choose the relative safety offered by vaccination against the well-known risks of the coronavirus and the associated disease. When it comes to vaccination more generally, there is conflicting evidence whether women are more likely than men to be vaccinated or to hold anti-vaccine attitudes (see overview in Ref.^10^).

While higher levels of vaccine hesitancy for women have been noted in different samples across the world, there is relatively little work that tries to ‘explain’ why this is the case and to account for the gender gap in vaccination attitudes.^3^ The main objective of this article is to propose an explanation of this phenomenon by examining a number of hypotheses suggested by existing academic literature and popular discussions.

One possible explanation of the gender gap is concerns about how COVID-19 vaccines affect and interact with conception, pregnancy and breastfeeding. Health authorities and doctors in several countries (e.g. Bulgaria) have issued warnings about interference of the vaccination with these practices, which—warranted or not—might have influenced the attitudes of people.^13^^,^^14^ Naturally, such concerns are likely to be higher among women, and younger women in particular. Relatedly, there is evidence for the effect of parenting role on vaccine hesitancy that is higher for women.^15^

Another hypothesis refers to the alleged higher use of women of online social networks for health-related information.^16^ Given that a lot of misinformation related to COVID-19 and vaccines has been spreading via online networks,^17^^,^^18^ it is possible that this can account for the gender gap in vaccine hesitancy.

Relatedly, it has been proposed that women are less likely to trust official health authorities and the medical profession because of perceived gender bias in these institutions.^19^ For many years, women have been largely excluded from the profession, have had lower rates of participation in clinical trials and medical studies more generally, so that medical advice and recommended practices are often geared toward men and ill-adapted to women. Such generalized mistrust of health authorities can undermine support for the vaccines promoted by official health authorities.

The final hypothesis we consider refers to different risk attitudes and preferences of men and women.^12^^,^^20^ This argument has two sides: perceived risks of COVID-19 and perceived risks and benefits of the vaccines. If women perceive lower risks of getting COVID-19 and lower severity and risk of death if they are infected by the virus (which would be justified based on clinical data^21^^,^^22^) this might result in higher vaccine hesitancy. But even if the COVID-19 risks are deemed high, different levels of hesitancy can appear from different assessments of the risks and benefits of vaccines, even if women and men are exposed to the same information. Yet, in 16 studies men showed lower perceived risk of getting COVID-19 and were not more concerned about the health consequences than women.^23^

Still, for women, the status quo before the vaccine development might be perceived as safer than vaccination based on hasty development of vaccines with unknown long-term consequences. There is some evidence that although women were not under-represented as participants in randomized controlled trials for vaccine effectiveness, there was insufficient reporting and analysis of COVID-19 vaccine data by gender and when disaggregated data were available, ‘the majority of participants reporting adverse events were women’.^24^^,^^25^ In an uncertain information environment surrounding the risks of vaccines, if men are more risk-‘accepting’ indeed, then this could account for their faster embrace of vaccines as a way to end the pandemic.

To test these hypotheses, the study uses data from two large comparative nationally representative surveys of public opinion fielded in the first half of 2021 in 27 European countries with a total of more than 50 000 respondents.

Understanding vaccine hesitancy and the gender gap in particular is important because these attitudes are strongly related to relevant behaviors, such as getting vaccinated or vaccinating one’s children. Even small delays in the decision to vaccinate induced by vaccine hesitancy can have important consequences at the societal level for the efforts of governments and public health authorities to contain the spread of the virus and limit the impact of the disease. Identifying the reasons why women are more likely to express vaccine hesitancy can also help designing targeted information campaigns to combat the phenomenon. To be sure, gender is but one demographic factor that is related to vaccine hesitancy: other relevant predictors have been identified, including age, education, occupation, but also social attitudes such as trust in authorities and many more (for recent overviews, see Refs^26–29^). But understanding the gender gap is important both for societal and for scientific reasons.

## Methods

This study is based on the analysis of two large comparative surveys of public opinion. The first survey (Standard Eurobarometer 94.3) covered 37 countries (from which we use the data on the 27 EU member states for comparability purposes with the second survey that we analyze) and was fielded at the request of the European Commission by Kantar.^30^ The sample size per country was between 1000 and 1100 respondents (median of 1040), with the exception of Luxembourg and Malta (which had between 500 and 600 respondents) and Germany (which had 1575). Data collection took place between 12 February 2021 and 18 March 2021. Multi-stage, random sampling procures were used, with the first-stage sampling points being administrative regional units. In principle, interviews took place in person using CAPI (Computer Assistance Personal Interviewing), but because of the coronavirus pandemic, in 15 countries the interviews were conducted online and in 5 more both face-to-face and online interviews were used. In most countries, online sampling was also probabilistic (based on a Random Digit Dialing design, the country telephone directories or proprietary panels).

The second survey (Flash Eurobarometer 494) covered the 27 member states of the EU and was fielded in May 2021 at the request of the European Commission by Ipsos European Public Affairs, Brussels. Sampling was based on quota-based nationally-representative samples.^31^ The interviews were web-based and conducted via self-administered questionnaires. The sample size for most countries was around 1000 respondents (only Malta, Cyprus and Luxembourg had a smaller number of respondents at around 515 each). Both surveys provide post-stratification weights based on age, region and degree of urbanization.

The main outcome variables of interest are COVID-19 vaccine hesitancy^32^ and vaccine refusal. Respondents are considered ‘vaccine hesitant’ if they responded that they will ‘Never’ get vaccinated against COVID-19 or will get vaccinated ‘Later’ (but not ‘some time in 2021’) or ‘Don’t know’ or prefer not to answer the question. (Excluding nonresponses does not affect the results significantly.) Vaccine refusal refers only to those who declare that they will ‘Never’ get vaccinated. The original formulation of the survey question was: ‘When would you like to get vaccinated against COVID-19 (coronavirus)?’ and the remaining answer categories, in addition to the ones mentioned above, are ‘As soon as possible’, ‘Some time in 2021’, and ‘I have already been vaccinated’. For the operationalization and measurement of the covariates used in the analyses, see the Supplementary material.

To examine how the gender gap evolves with age, we use binomial generalized additive models with logic link,^33^ with the probabilities of vaccine hesitancy and refusal estimated separately for each gender and age entered as a smooth term. To examine how the other factors related to our hypotheses influence the gender gap, we rely on chi-squared tests and multivariate logistic regression models.

## Results

**Figure 1:**
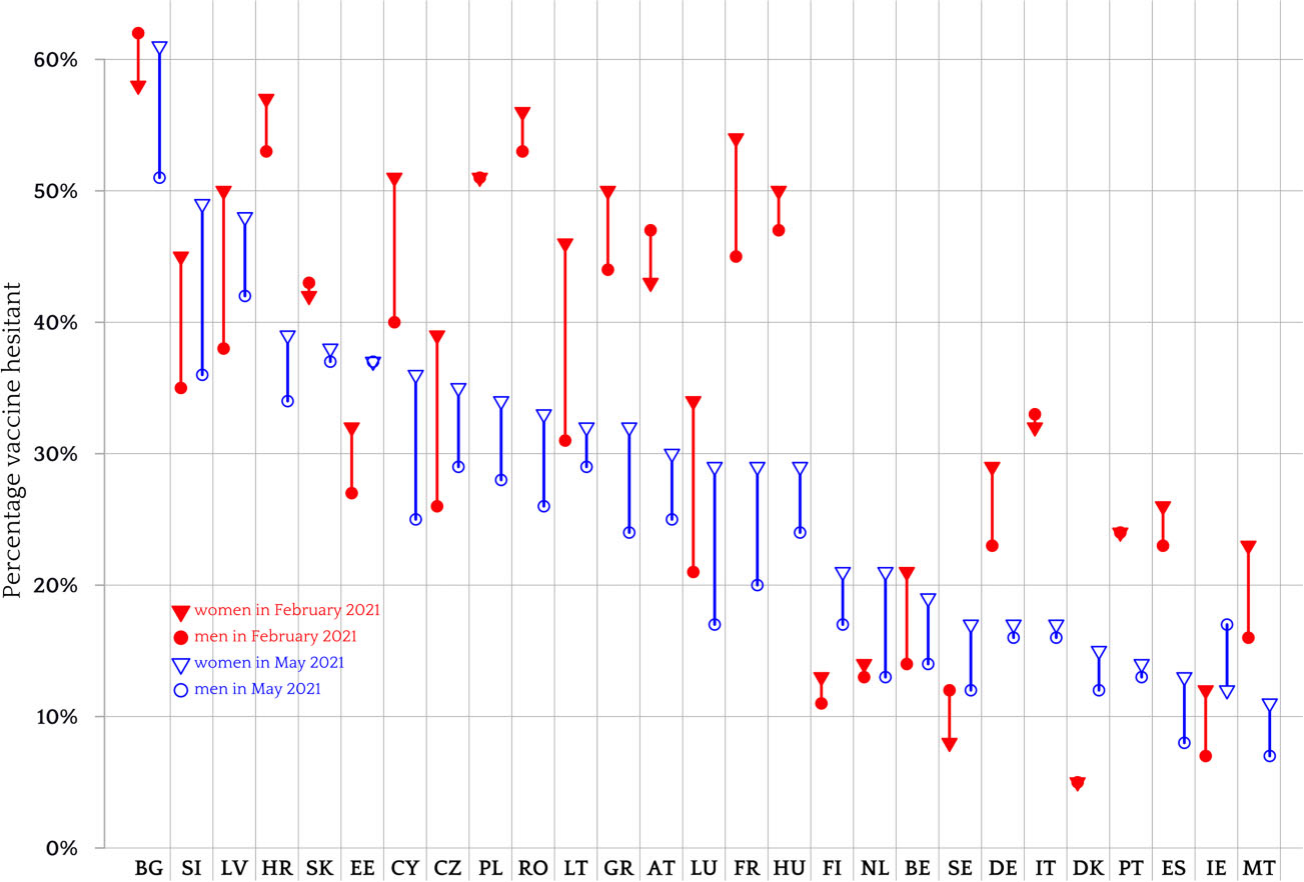
Percentage of women (triangles) and men (dots) who express COVID-19 vaccine hesitancy in February 2021 (solid) and in May 2021 (empty) in each of the 27 member states of the EU

In February 2021, 36.1% of women expressed vaccine hesitancy vs. 30.8% in the sample of respondents from the EU-27 (for the Pearson’s chi-squared χ^2^ test statistics and associated *P*-values, see Supplementary table SA1). In May 2021, 28.7% of women vs. 23.5% of men expressed vaccine hesitancy. Therefore, while vaccine hesitancy declined significantly in the first part of 2021, the gender gap remained. The gender gaps with respect to vaccine refusal were smaller—13.8% vs. 11.6% in February and 12.0% vs. 10.5% in May—but the differences between women and men were still significant. As further evidence to the systematic nature of the gender gap, women were more likely to be vaccine hesitant than men in 22 out of 27 countries in February 2021 (the exceptions were Slovakia, Italy, Austria, Sweden and Bulgaria) and in 26 out of 27 countries in May 2021 (Ireland was the only exception). In four countries—Slovenia, Luxembourg, Cyprus and Bulgaria—the gap was 10 percentage points or wider. Figure 1 shows the distribution of vaccine hesitancy per gender, for each country and for the two time periods.

**Figure 2:**
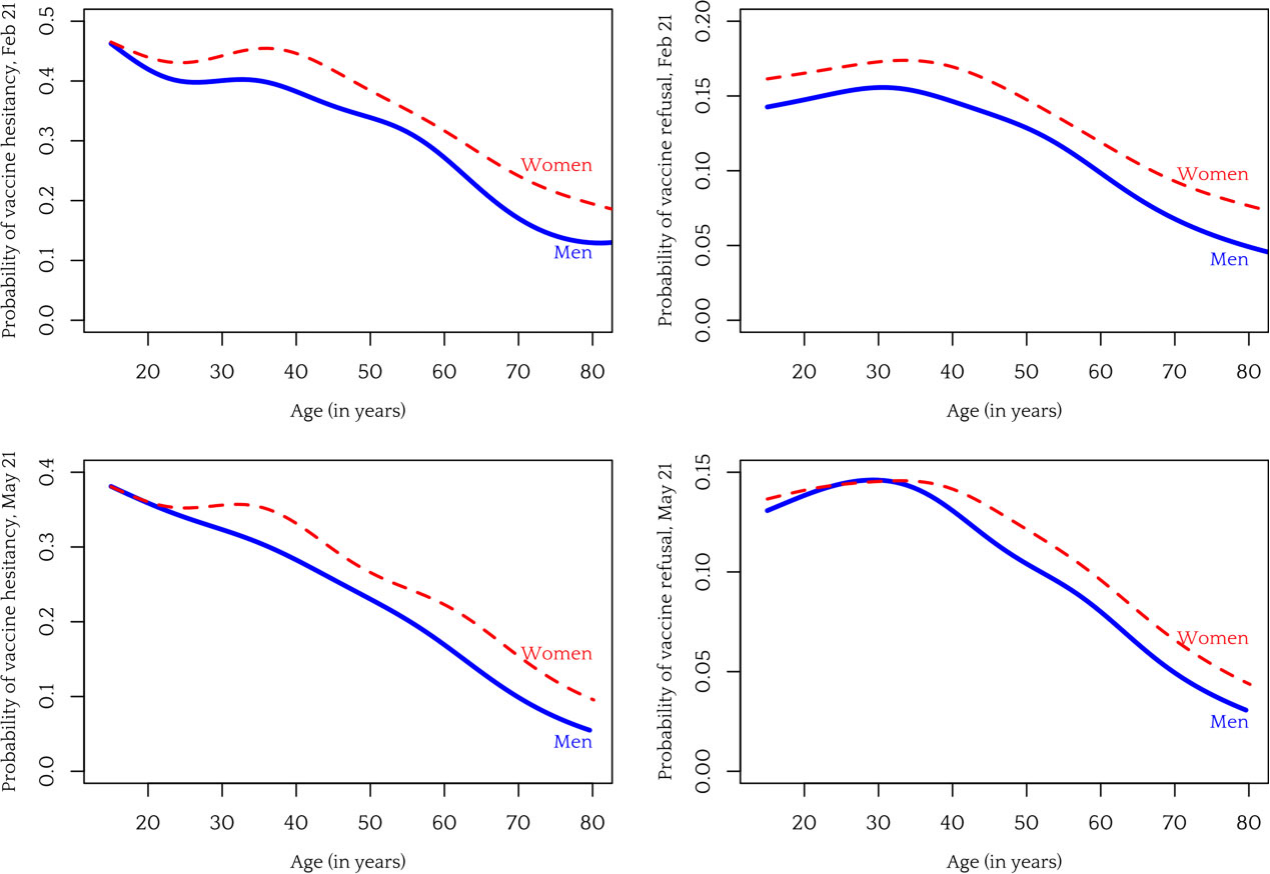
Probability of COVID-19 vaccine hesitancy (left) and vaccine refusal (right) as a function of age, per gender. Top row shows data from February 2021, bottom row from May 2021. Estimates based on binomial generalized additive models with logit link and smooth term for age

The first hypothesis we consider is that the gender gap results from concerns related to conception, pregnancy and breastfeeding. If such concerns are a major reason for the vaccine hesitancy gap, we would expect that the gap disappears as women pass fertility age. Figure 2 shows the results of nonparametric estimation of the effect of age on vaccine hesitancy in May 2021.

As we can see from the plots in figure 2, there is no evidence that the gender gap disappears or even declines as women pass fertility age. Very young women and men differ less in their probability of vaccine hesitancy and refusal (in May 2021), but, once it opens, the gender gap persists past the age when pregnancy and breastfeeding should be major personal concerns for women.

Next, we consider the hypothesis that women have lower trust in health institutions and professionals due to a long history of male dominance in related professions and underrepresentation of women in medical studies. In February 2021, women were ‘more’ likely to express trust in ‘health and medical staff’ in their country: 83.9% vs. 82.7 for men. Comparing the levels of trust women and men put into different sources of ‘reliable information on COVID-19 vaccines’ in particular, we find only minor differences in May 2021. Women trusted health authorities at 43.4%, while men did at 44.2%. Women trusted doctors and other medical professionals (for COVID-19 vaccine information) at 62%, which was actually higher than the level for men (59%). It is remarkable that trust in health authorities (53.8% women; 53.3% men) and medical professionals (74.5% women; 69.8% men) as sources of trustworthy COVID-19 vaccine information ‘declined’ between February and May 2021, just as vaccine acceptance ‘increased’ during the same period.

Next, we consider the hypothesis that the gender gap results from higher trust of women in social networks and the Internet as sources of medical information. In February 2021, women reported higher general trust in online social networks (22.0% vs. 20.4% for men), but not in the Internet as a media (40.0% vs. 40.3%). In May 2021, women were in fact less likely to report that they trust the Internet (7.3% vs 8.7% for men) and online social networks (5.3% vs. 6.1% for men) as sources of reliable information on COVID-19 vaccines, in particular. They were, however, more likely to trust ‘people around you (colleagues, friends and family)’ (15.9% vs. 15.0% for men), which is a significant predictor of vaccine hesitancy.

Next, we turn to the hypothesis that the gender gap can be explained by reference to the different risks and benefits women perceive from COVID-19 and from the vaccines. Women were actually more likely to fear getting infected with COVID-19 in the future (in May 2021): women 45.6%, men 40.6. Furthermore, women were ‘not’ more likely to believe that one can avoid COVID-19 without vaccination: women 47.4%, men 48.2%, χ^2^ = 1.63; *P* values = 0.20.

With regard to beliefs about the risks and benefits of vaccines, there are clear gender differences. Women were significantly more likely to agree that COVID-19 vaccines were developed too quickly to be safe (in February 2021, women 56.4%, men 50.4%; levels of agreement with this statement ‘increased’ in May 2021, but the gender gap remained just as large). Similarly, women were significantly more likely to agree that COVID-19 vaccines could have unknown long-term side effects (in February 2021, women 70.3%, men 63.8%; levels were slightly lower in May 2021, but the gender gap increased slightly to 8 percentage points). They were also less likely to agree that a vaccine is the only way to stop the pandemic (in February 2021, women 69.7%, men 72.4; the gap increased slightly in May 2021).

When asked about vaccines in general, women expressed lower levels of agreement that vaccines are safe (women 75.0%, men 80.0%) and effective (women 83.0%, men 85.1%). With respect to the belief that ‘vaccines authorised in the European Union are safe’, the gender gap was even wider (women 65.7%, men 72.5%, χ^2^ = 143.35; *P* values < 0.001). In line with this pattern, women were less likely to agree that ‘all in all, benefits of COVID-19 vaccines outweigh possible risks’ (women 70.1%, men 76.2%).

Table 1 shows the results from five logistic regression models (with logit link): Model 1 has only the gender variable as a predictor and serves as a baseline. Model 2 adds demographic variables and country indicators (the 26 country coefficients per model are not printed as they are not of substantive interest); Model 3 adds trust in various sources of COVID-19 information; Model 4 adds COVID-19 and vaccine-related beliefs; and Model 5 adds only one additional variable: agreement that vaccines have more benefits than risks. The coefficients reported in the table are unexponentiated (the table shows the log of the odds ratios), so that a negative sign implies lower probability of vaccine hesitancy and a positive sign implies a higher probability of vaccine hesitancy.

**Table 1:** Logistic regression models of vaccine hesitancy (*Data*: Eurobarometer from May 2021)

Variable	Model 1		Model 2		Model 3		Model 4		Model 5	
	log (OR)	*P*-value	log (OR)	*P*-value	log (OR)	*P*-value	log (OR)	*P*-value	log (OR)	*P*-value
Gender (male)	−0.27	**<0.001**	−0.29	**<0.001**	−0.26	**<0.001**	−0.24	**<0.001**	−0.15	**0.002**
Age			−0.03	**<0.001**	−0.03	**<0.001**	−0.03	**<0.001**	−0.02	**<0.001**
Education			−0.03	**<0.001**	−0.02	**<0.001**	−0.01	0.20	−0.01	**0.10**
Occupation										
Employee			–		–		–		–	
Manual worker			0.53	**<0.001**	0.42	**<0.001**	0.35	**<0.001**	0.28	**0.003**
No activity			0.24	**<0.001**	0.29	**<0.001**	0.33	**<0.001**	0.32	**<0.001**
Self-employed			0.29	**<0.001**	0.21	**<0.001**	0.14	**0.044**	0.14	**0.050**
Residence (city)			−0.22	**<0.001**	−0.16	**<0.001**	−0.14	**0.003**	−0.13	**0.008**
Trust.EU.info					−0.95	**<0.001**	−0.63	**<0.001**	−0.49	**<0.001**
Trust.gov.info					−0.63	**<0.001**	−0.32	**<0.001**	−0.17	**0.033**
Trust.health.info					−1.1	**<0.001**	−0.61	**<0.001**	−0.41	**<0.001**
Trust.local.info					−0.43	**<0.001**	−0.40	**<0.001**	−0.38	**<0.001**
Trust.doctors.info					−0.86	**<0.001**	−0.43	**<0.001**	−0.33	**<0.001**
Trust.media.info					−0.39	**<0.001**	−0.14	**0.090**	−0.08	0.35
Trust.web.info					0.29	**<0.001**	0.16	**0.039**	0.10	0.26
Trust.networks.info					0.46	**<0.001**	0.24	**0.010**	0.17	**0.078**
Trust.people.info					0.05	0.30	−0.07	0.21	0.00	0.96
Vaccines are safe							−0.86	**<0.001**	−0.52	**<0.001**
Vaccines effective							−1.1	**<0.001**	−0.82	**<0.001**
Fears COVID-19 infection							−0.90	**<0.001**	−0.70	**<0.001**
COVID-19 can be avoided							0.78	**<0.001**	0.68	**<0.001**
Vaccines developed too fast							0.92	**<0.001**	0.79	**<0.001**
Vaccines unknown side effects							0.80	**<0.001**	0.80	**<0.001**
Vaccines more benefits than risks									−1.9	**<0.001**
AIC	29 954		20 012		17 508		13 799		12 296	
*N* observations	26 106		19 944		19 944		19 944		19 944	

*P*-values equal to or lower than 0.10 are in bold.

According to the baseline Model 1, men are significantly less likely to be vaccine hesitant than women. Adding demographic variables even increases slightly the gender gap. Adding the trust-related variables does little to reduce the effect of gender, although many of these variables have significant effects as such. Even when we add relevant COVID-19 and vaccine-relevant beliefs, the coefficient of gender drops in size only from −0.27 to −0.24, which makes for a small decrease in the size of the gender gap in vaccine hesitancy, conditional on all these variables. Remarkably, when a single additional variable is added in Model 5—the belief that the benefits of vaccines outweigh the associated risks, the effect of gender drops significantly to −0.14. The fit of Model 5 is also significantly better than the rest.

When the same set of models are estimated using vaccine refusal as the outcome variable, the effect of gender is smaller to begin with, and disappears completely once we include the variables related to vaccine beliefs (see Supplementary table SA2). This suggests that the higher vaccine hesitancy among women is not due to fundamental opposition to vaccination, but to higher levels of caution, in line with the results reported above.

There are some significant differences in the reasons men and women endorse for and against vaccination. Women were less likely to agree that vaccination is a civic duty (women 52.9%, men 61.3%) and to support compulsory vaccination (women 39.0%, men 45.9%). Among the vaccine hesitant respondents, women were less likely to be against vaccines in general, to say that the infection risk is too low for them personally, to think that the pandemic will be over soon anyway, and—importantly—to agree that the risks of COVID-19 are exaggerated. However, women were more likely to be worried about side effects and to think that vaccines are not effective (see Supplementary figures SA1 and SA2). (Note that these questions were asked only to vaccine hesitant respondents, so they do not allow us to make inferences about the prevalence of these attitudes among all women and all men.)

## Discussion

Using data from two large comparative surveys of public opinion in 27 European countries from the first half of 2021, this article showed that a significant gender gap existed with respect to COVID-19 vaccine hesitancy and, to a lesser extent, vaccine refusal. The hypotheses that the gap results from (i) pregnancy, breastfeeding and fertility concerns, (ii) higher trust in Internet and social networks as sources of medical information, (iii) lower trust in health authorities and (iv) lower perceived risks of getting infected with COVID-19 did not find support from the statistical tests and analyses conducted. The one hypothesis that is consistent with the data is that women were more likely to perceive COVID-19 vaccines as unsafe and ineffective and this leads them to consider the net benefits of vaccination as lower than the associated risks. The last factor appears to be the most important one behind the gender gap in COVID-19 vaccination attitudes.

The analysis does not find that the gender gap declines with age. While it is possible that once women develop concerns about a particular type of vaccine, such concerns could spill over into old age, this is less likely to be the case with COVID-19 vaccines, which have been developed very recently. The fact that the gender gap is evident as early as February 2021, before any reliable information about gender-specific side effects could have been present, also speaks against the idea that concrete gender-specific health concerns were responsible for the gap in attitudes.

Overall, there is no evidence that women are more likely to derive trustworthy information from the Internet and online networks for vaccine-relevant information, which is not consistent with the expectations of the hypothesis we consider. We have no evidence to conclude that women perceived lower risks of getting infected with COVID-19 (in line with Ref.^23^), although we have no data as to how grave they thought the consequences of getting infected would be.

The most significant differences between genders that we observe are with regard to beliefs about the risks and benefits of vaccines, with women being much more likely to express concern and consider that the risks outweigh the potential benefits. These results are consistent with the hypothesis that differential risk perceptions account for the gender gap in COVID-19 vaccination attitudes, although with the important qualification that women perceive risks from COVID-19 as higher as well; just the risks of vaccines are perceived ‘much higher’ than among men.

The differential assessment of potential risks and benefits of the COVID-19 vaccine appeared quite early in 2021, before concrete and reliable information was available about possible side effects. This suggests that the differential assessment might be due to a form of ‘status quo bias’ that is more prevalent among women^34^ and interacts with risk aversion.

The study has important limitations related to its temporal and geographical focus, which was limited to European countries only. But the evidence for a gender gap in vaccination attitudes from across the world suggests that the causal structure behind it might be similar as well to the one outlined in this article for the European case.

Both surveys analyzed in this article were fielded in the first half of 2021, when the mass vaccination campaigns were being rolled out in Europe and not much was known yet about the real-world effectiveness and possible side effects of the vaccines. It is possible that the gender gap and the reasons behind it changed with time, but our results still remain substantively relevant and important. Even if strong government incentives for COVID-19 vaccinations reduced vaccine hesitancy and closed the gender gap later in 2021 and 2022, related attitudes, such as those toward the vaccination of children or getting booster vaccination shots, likely have similar antecedents. In this respect the relevance of this study goes beyond the immediate empirical context of early-stage COVID-19 vaccination campaigns. One of the important open questions that remain is to what extent vaccine attitudes determine behavior and whether hesitancy can be overcome with targeted information campaigns, for example. But even if attitudes just ‘delay’ vaccination, the public health consequences can be considerable.

In this respect, the results of this study suggest that information campaigns might need to be gender-specific. If women are more likely to assume that the risks of vaccination outweigh the benefits even in the absence of strong evidence to this effect, messages need to be crafted to address such concerns early on in public health campaigns. Some existing research, however, does not find strong gender-specific effects of information messages on the role of COVID-19 vaccination.^3^^,^^7^^,^^35^ Yet, the fact that we do not find support for the ideas that the gender gap results from structural factors, such as systematically lower trust of health authorities or higher use of social networks as sources of medical information, implies that communication can be effective in closing the gender gap. In this respect, even if gender is only one among many predictors of vaccine hesitancy and refusal (see, i.e. Refs^27^^,^^29^), it is one that deserves further study.

It should be reminded that the data for the current analyses come from public opinion surveys using web-based data collection. It could be that the mode of data collection provides a biased estimate of the overall level of vaccine hesitancy and refusal. More generally, irrespective of the mode of data collection, public opinion surveys might underestimate the level of vaccine hesitancy due to respondents holding such views being less likely to participate or reveal their preferences truthfully.^36^ Most importantly, however, we have no reason to believe that such biases—if present—work differently for men and women, so the estimates of the effects of different variables on the gender bias in vaccine hesitancy should still be valid. Nevertheless, future research could use population-based vaccine registration data to study the same phenomena without the threat of response bias.

## Supplementary Material

ckad052_Supplementary_Data

## Data Availability

The data used in this article are available on the GESIS archive: European Commission, Brussels (2023). *Eurobarometer 94.3 (2021)*. GESIS, Cologne. ZA7780 Data file Version 2.0.0, https://doi.org/10.4232/1.14076. European Commission, Brussels (2022). *Flash Eurobarometer 494 (Attitudes on Vaccination against Covid-19)*. GESIS, Cologne. ZA7771 Data file Version 2.0.0, https://doi.org/10.4232/1.14030. The R script analyzing the data is available at: https://github.com/demetriodor/gender-gap-vaccination-attitudes.
