# Supplementary material for: Explaining the gender gap in COVID-19 vaccination attitudes
Source: Eur J Public Health. 2023 May 13;33(3):490–5. doi: 10.1093/eurpub/ckad052 (PMC10234652; doi:10.1093/eurpub/ckad052)
Supplement: ckad052_Supplementary_Data [file ckad052_supplementary_data.docx]

# Online supplementary material

## Operationalization and measurement of the covariates used in the analysis

The main demographic predictors of interest were age (measured in years), education (measured as the age at which full-time education was stopped), gender, place of residence (‘Large town/city’ vs. ‘Small or medium-sized town’ and ‘A rural area of village’), and professional occupation (with four categories: ‘Self-employed’, ‘Employee’, ‘Manual worker’, and ‘Without a professional activity').

Trust in authorities was measured with the question: ‘How much trust do you have in certain institutions? For each of the following institutions, do you tend to trust it or tend not to trust it?’ with respect to ‘Health and medical staff in (OUR COUNTRY)’.

General trust in media was measured with the question: ‘How much trust do you have in certain media? For each of the following media, do you tend to trust it or tend not to trust it?’ with respect to ‘The internet’ and ‘Online social networks’.

Trust in different authorities with respect to COVID-19-related information was measured via the question ‘Among the following sources, which ones would you trust more to give you reliable information on COVID19 vaccines?’, with multiple answers possible from the following categories: ‘The European Union’, ‘The National Government’, ‘The National Health Authorities’, ‘The regional or local public authorities’, ‘Health professionals, doctors, nurses and pharmacists’, ‘Media (television, radio, newspapers’, ‘Websites’, ‘Online social networks’, ‘People around you (colleagues, friends and family)’ and ‘Don’t know’).

Several vaccine-related beliefs were measured: ‘Vaccines are safe’, ‘Vaccines are effective’, ‘I believe vaccines authorised in the European Union are safe’, ‘All in all, benefits of COVID-19 vaccines outweigh possible risks’, ‘COVID-19 vaccines are being developed, tested and authorised too quickly to be safe’, ‘COVID-19 vaccines could have long term side-effects that we do not know yet’, ‘A vaccine is the only way to end the pandemic’, ‘You can avoid being infected by COVID-19 without being vaccinated’, ‘COVID-19 vaccination should be compulsory’ and ‘Everyone should get vaccinated against COVID-19, it is a civic duty’. Originally, answers to all these were recorded on 4-point agreement scales, which were collapsed in two categories (‘Agree totally or tend to agree’ vs. ‘Disagree totally or tend to disagree’). An additional item related to knowledge about COVID-19 was used: whether the respondent fears to be infected with COVID-19 in the future [yes/no answer].

***Table A1****.* Pearson's chi-squared χ2 test statistics (and p-values) of the differences in vaccine hesitancy and refusal and related attitudes and beliefs between men and women (Data: Eurobarometer from February/March 2021 and May 2021)

| **Variable** | **February 2021** | | **May 2021** | |
| --- | --- | --- | --- | --- |
|  |  |  |  |  |
| Vaccine hesitancy | 86.19 (<0.001) | | 89.47 (<0.001) | |
| Vaccine refusal | 29.96 (<0.001) | | 15.35 (<0.001) | |
| Trust in health and medical staff | 6.64 (<0.01) | |  | |
| Trust in health authorities |  | | 1.78 (0.18) | |
| Trust in doctors and other medical professionals |  | | 24.97 (<0.001) | |
| Trust in online social networks | 10.46 (<0.01) | |  | |
| Trust in Internet as a media | 0.18 (0.67) | |  | |
| Trust in people around you |  | | 4.62 (0.03) | |
| Fear of getting infected with COVID-19 |  | | 63.72 (<0.001) | |
| One can avoid COVID-19 without vaccination | 101.02 (<0.001) | |  | |
| COVID-19 vaccines could have unknown long-term side effects | 132.02 (<0.001) | |  | |
| A vaccine is the only way to stop the pandemic | 24.24 (<0.001) | |  | |
| Vaccines are safe | 97.27 (<0.001) | |  | |
| Vaccines are effective | 22.84 (<0.001) | |  | |
| Vaccines authorised in the European Union are safe | 143.35 (<0.001) | |  | |
| All in all, benefits of COVID-19 vaccines outweigh possible risks | 122.02 (<0.001) | |  | |
| Vaccination is a civic duty |  | | 186.96 (<0.001) | |
| Support compulsory vaccination |  | | 126.28 (<0.001) | |

***Table A2.*** Logistic regression models of vaccine refusal (Data: Eurobarometer, May 2021)

| **Variable** | **Model 1** | | **Model 2** | | **Model 3** | | **Model 4** | | **Model 5** | |
| --- | --- | --- | --- | --- | --- | --- | --- | --- | --- | --- |
|  | **log(OR)***^1^* | **p-value** | **log(OR)***^1^* | **p-value** | **log(OR)***^1^* | **p-value** | **log(OR)***^1^* | **p-value** | **log(OR)***^1^* | **p-value** |
| Gender *[male]* | -0.15 | <0.001 | -0.16 | <0.001 | -0.10 | 0.046 | -0.06 | 0.27 | 0.05 | 0.44 |
| Age |  |  | -0.02 | <0.001 | -0.02 | <0.001 | -0.02 | <0.001 | -0.02 | <0.001 |
| Education |  |  | -0.03 | <0.001 | -0.01 | 0.046 | 0.00 | 0.56 | -0.01 | 0.32 |
| Occupation |  |  |  |  |  |  |  |  |  |  |
| *Employee* |  |  | — |  | — |  | — |  | — |  |
| *Manual worker* |  |  | 0.31 | 0.001 | 0.12 | 0.23 | 0.02 | 0.85 | -0.03 | 0.83 |
| *No activity* |  |  | 0.21 | <0.001 | 0.24 | <0.001 | 0.24 | 0.001 | 0.25 | 0.001 |
| *Self-employed* |  |  | 0.34 | <0.001 | 0.26 | <0.001 | 0.19 | 0.024 | 0.19 | 0.026 |
| Residence *[city]* |  |  | -0.21 | <0.001 | -0.13 | 0.015 | -0.12 | 0.056 | -0.11 | 0.090 |
| Trust.EU.info |  |  |  |  | -1.4 | <0.001 | -1.0 | <0.001 | -0.79 | <0.001 |
| Trust.gov.info |  |  |  |  | -0.74 | <0.001 | -0.26 | 0.035 | -0.02 | 0.88 |
| Trust.health.info |  |  |  |  | -1.4 | <0.001 | -0.92 | <0.001 | -0.68 | <0.001 |
| Trust.local.info |  |  |  |  | -0.57 | <0.001 | -0.40 | 0.005 | -0.34 | 0.022 |
| Trust.doctors.info |  |  |  |  | -1.2 | <0.001 | -0.75 | <0.001 | -0.67 | <0.001 |
| Trust.media.info |  |  |  |  | -0.56 | <0.001 | -0.19 | 0.11 | -0.06 | 0.61 |
| Trust.web.info |  |  |  |  | 0.30 | <0.001 | 0.18 | 0.064 | 0.12 | 0.22 |
| Trust.networks.info |  |  |  |  | 0.51 | <0.001 | 0.39 | <0.001 | 0.35 | 0.002 |
| Trust.people.info |  |  |  |  | -0.31 | <0.001 | -0.42 | <0.001 | -0.32 | <0.001 |
| Vaccines are safe |  |  |  |  |  |  | -0.68 | <0.001 | -0.38 | <0.001 |
| Vaccines effective |  |  |  |  |  |  | -0.82 | <0.001 | -0.64 | <0.001 |
| Fears COVID-19 infection |  |  |  |  |  |  | -1.2 | <0.001 | -1.0 | <0.001 |
| COVID-19 can be avoided |  |  |  |  |  |  | 0.91 | <0.001 | 0.79 | <0.001 |
| Vaccines developed too fast |  |  |  |  |  |  | 0.67 | <0.001 | 0.51 | <0.001 |
| Vaccines unknown side effects |  |  |  |  |  |  | 0.95 | <0.001 | 0.92 | <0.001 |
| Vaccines more benefits than risks |  |  |  |  |  |  |  |  | -1.7 | <0.001 |

**
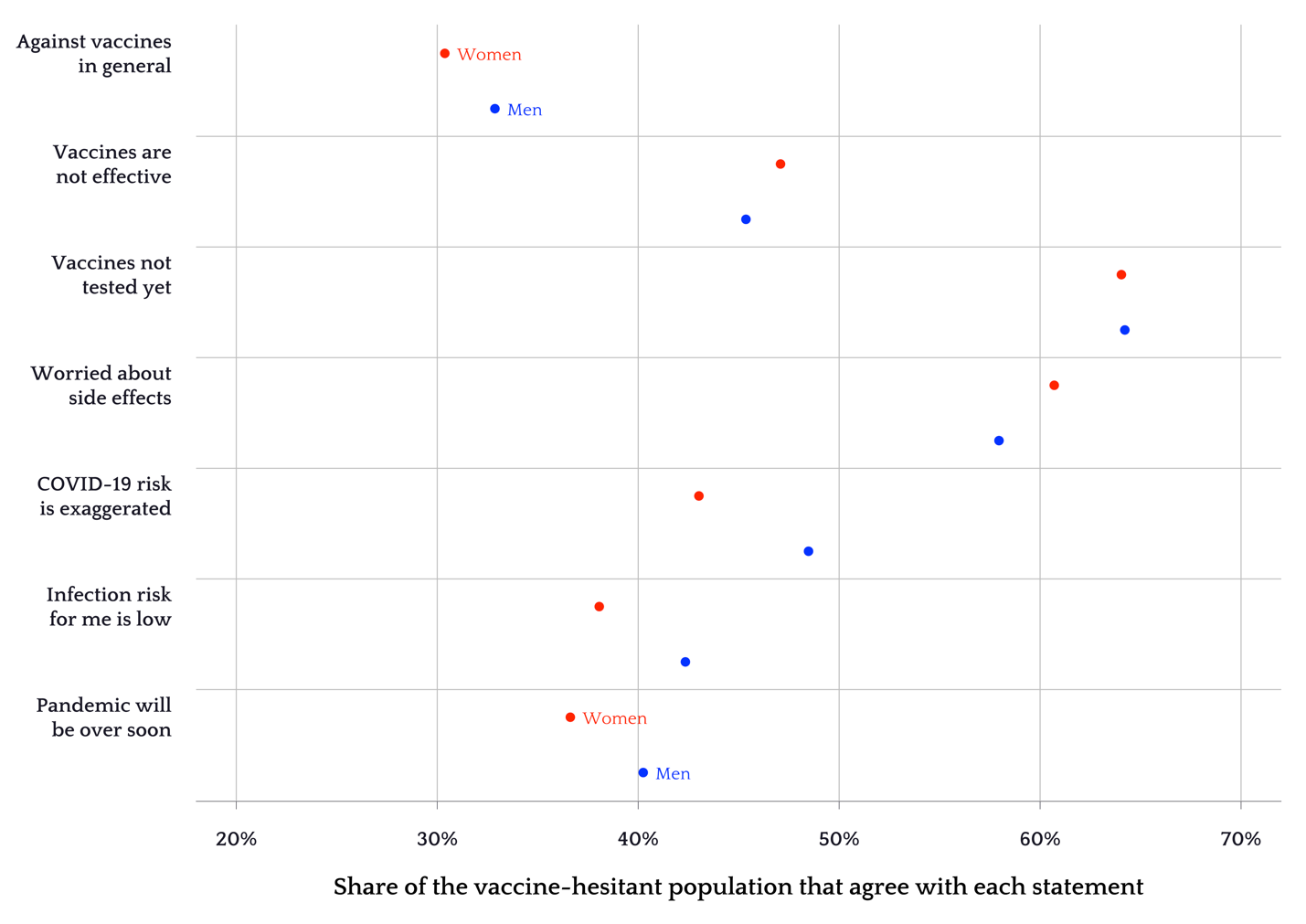
Figure A1.** Percentage of women (red dots) and men (blue dots) who endorse a particular reason (shown on the y-axis) for COVID-19 vaccine hesitancy, from those who are vaccine hesitant, May 2021


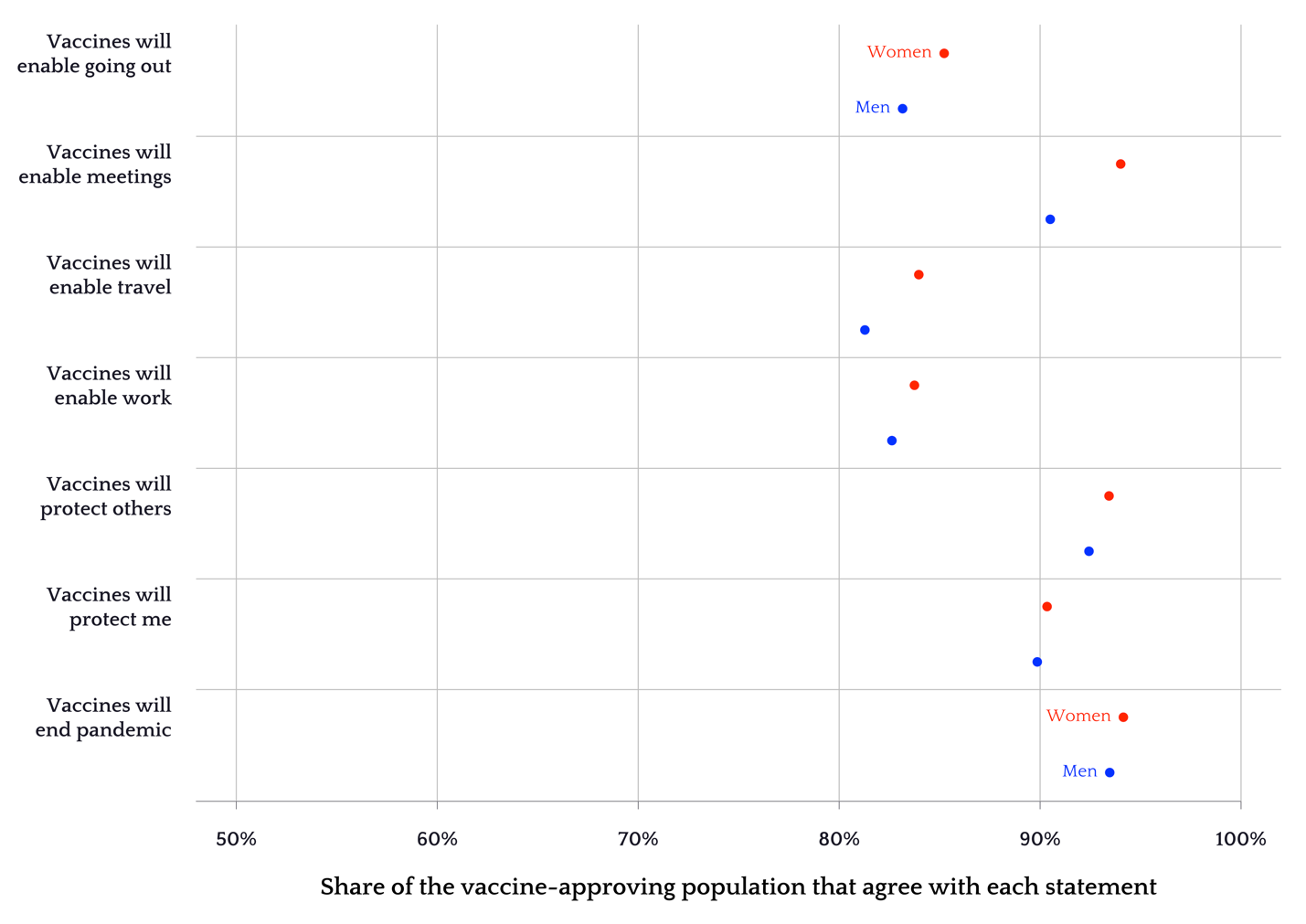


**Figure A2.** Percentage of women (red dots) and men (blue dots) who endorse a particular reason (shown on the y-axis) for COVID-19 vaccine acceptance, from those who are vaccine accepting, May 2021
